# New parajeilongviruses detected in bats but not in humans: assays for screening and diagnostic purposes

**DOI:** 10.1007/s00705-025-06520-1

**Published:** 2026-01-15

**Authors:** Emilia Pulkkinen, Reilly Jackson, Ruut Joensuu, Essi M. Korhonen, Moses Muia Masika, Omu Anzala, Joseph G. Ogola, Paul W. Webala, Tamika J. Lunn, Kristian M. Forbes, Olli Vapalahti, Tuure Kinnunen, Tarja Sironen, Anne J. Jääskeläinen

**Affiliations:** 1 HUS Diagnostic Center, Clinical Microbiology, Helsinki University Hospital and University of Helsinki, Helsinki, Finland; 2 ISLAB Laboratory Centre, Kuopio, Finland; 3 Department of Virology, University of Helsinki, P.O. Box 21, Helsinki, FI-00014 Finland; 4 Department of Veterinary Biosciences, University of Helsinki, P.O. Box 66, Helsinki, FI-00014 Finland; 5 University of Eastern Finland, Kuopio, Finland; 6 KAVI Institute of Clinical Research, University of Nairobi, Nairobi, Kenya; 7 Department of Medical Microbiology, Faculty of Health Sciences, University of Nairobi, Nairobi, Kenya; 8 Department of Biological Sciences, University of Arkansas, Fayetteville, AR US; 9 Department of Forestry and Wildlife Management, Maasai Mara University, Narok, Kenya

**Keywords:** Bat, Diagnostics, Emerging viruses, Henipavirus, Jeilongvirus, Paramyxoviruses, RT-PCR, Zoonosis

## Abstract

To enhance preparedness against existing and new zoonotic viruses such as Hendra virus, Nipah virus, and other paramyxoviruses, screening tools and efficient diagnostic methods are needed. Here, we established a conventional nested pan-PCR assay using previously described primers for screening of human and bat samples. Additionally, we developed specific real-time RT-PCR (RT-qPCR) assays to detect Nipah virus and Hendra virus genotypes 1 and 2. Both PCR methods demonstrated good performance and could be used for screening of paramyxoviruses. Human serum and cerebrospinal fluid samples from 558 Finnish patients and 60 serum samples from Kenyan patients were screened using the nested-pan-PCR assay, and all were negative. In addition, we screened 340 synanthropic bat samples collected during 2021 and 2023 from Kenya, resulting in the discovery of two parajeilongviruses in Angolan free-tailed bat (*Mops condylurus*) samples.

**Supplementary Information:**

The online version contains supplementary material available at 10.1007/s00705-025-06520-1.

## Introduction

Comprehensive preparedness is essential for a rapid response to emerging zoonotic pathogens and future pandemics. Research shows that 60.3% of emerging pathogens have a zoonotic origin, 71.8% of which originate from wildlife [1]. One of the families of high-risk viruses is *Paramyxoviridae*, in particular, the subfamilies *Orthoparamyxovirinae* and *Feraresvirinae*, which both deserve special attention when preparedness is established. These two subfamilies include diverse paramyxoviruses (PMVs) that infect mammals of various species. A notable PMV that infects humans is measles virus (*Morbillivirus hominis*). This virus spreads extremely efficiently via droplets and still continues to cause childhood mortality worldwide. Measles virus can also cause a wide spectrum of non-fatal complications, including central nervous system (CNS) infections and pneumonia [2]. Less-severe and more-common human PMV infections include respiratory tract infections caused by parainfluenza viruses.

Many PMVs possess zoonotic potential because of their high mutation rate, a typical characteristic of RNA viruses, and their ability to switch host species [3]. Two examples of such zoonotic viruses are the bat-borne viruses Hendra virus (HeV) and Nipah virus (NiV), which have caused severe, often fatal infections in humans in Southeast Asia and Australia [4, 5]. Fruit bats serve as the primary reservoirs for these viruses [6]. However, domesticated animals, such as horses and pigs, have been implicated as intermediate hosts facilitating virus transmission to humans [5]. HeV infections typically manifest as equine-borne sporadic spillover events in Australia, whereas NiV causes annual outbreaks linked to the consumption of contaminated date palm sap or through pigs, for example, in Bangladesh and India [7, 8]. A new emerging paramyxovirus with the pathogenicity of a henipavirus and the transmissibility of measles virus would pose a severe public-health risk, potentially triggering an epidemic – or even a pandemic.

Fruit bats are known to host various paramyxoviruses [9, 10]. For example, in Africa, henipavirus RNAs have been detected in straw-colored fruit bats (*Eidolon helvum*) in Ghana [11] and the Republic of Congo [12]. Moreover, serological evidence suggests the presence of henipa-like viruses circulating in domesticated pigs from several different pig farms in Uganda [13]. While no henipavirus infections in humans have been reported in Africa, Sosuga virus – a closely related pathogen belonging to the subfamily *Rubulavirinae* that is in *Rousettus aegyptiacus* bats in Uganda – has been transmitted to a human, causing acute febrile illness [14]. In Europe, PMVs have been detected in different mammalian species, including insectivorous bats, shrews, and hedgehogs [15–18]. However, spillover events from reservoir animals to humans have not been reported to date. The bat populations in Finland consist only of insectivorous bats [19], and it is currently unknown whether these bat species would carry potentially zoonotic PMVs. To date, no zoonotic PMV infections have been reported in Finland.

In order to study human samples collected from patients in Finland and Kenya with suspected CNS infections or other acute or febrile infections, we established a nested pan-PCR assay using previously designed primers [20] for the detection of PMVs. This assay was also used to screen bat samples from Kenya for the presence of PMVs. As nested assays are prone to contamination, i.e., due to cross-contamination from the positive PCR control, we designed a PCR control with a built-in modification that can be identified by sequencing to avoid false positives in diagnostic settings. For emergency settings, the nested pan-PCR assay is too slow and laborious to be practical. Accordingly, we established fast and specific RT-qPCR methods for the detection and discrimination of NiV and HeV. The HeV RT-qPCR was designed to detect both HeV genotype 1 (G1) and the recently discovered genotype 2 (G2) [21], in order to cover all of the viruses known to cause human disease.

## Materials and methods

### Human samples

Altogether, 679 samples from Finnish and Kenyan patients were collected and screened. From Finland, 606 serum samples and 13 cerebrospinal fluid samples were collected retrospectively from 558 individuals (median age, 46 years; range, 1–88 years; sex, data not available) between August 2018 and November 2018 from 16 different hospital districts and the Åland Islands. The samples were collected from anonymized sample archives with limited patient data. All samples were from patients with suspected severe acute viral infections, particularly central nervous system infections. Symptoms and differential diagnostics from Finnish patients are listed in Table 1. All data were anonymized, and the research was carried out with ethical approval (permits HUS/32/2018, HUS/211/2020, HUS/244/2021, and 159/HUS/151/2022) from the HUS Diagnostic Center, Helsinki, Finland. A subset of 60 serum samples from 60 Kenyan patients (median age, 19 years; range, 7 months-83 years; sex, 30 female, 29 male, 1 unknown) was obtained from a larger sample collection [22] from Taita-Taveta County, southeastern Kenya, in October 2016. All patients had an acute febrile illness, and the symptoms and differential diagnostics are listed in Table 2. Most of the Kenyan patients had been in contact with either domesticated farm animals or wild animals, including bats and rodents. The characteristics of the participants were reported previously by Masika et al. [22].

**Table 1:** List of differential diagnostics and symptoms of the Finnish patients (N = 558). The list includes tested viral and bacterial agents, and any data available derived from the laboratory data management system. Within approved research permits, only data available in the laboratory data management system could be exploited. No clinical diagnoses of the patients were available

**Individuals with a CSF sample screened for HSV1, HSV2, or VZV nucleic acids**				**81**
Positive for HSV2				1
Positive for VZV				2
**Individuals screened for enterovirus nucleic acids**				**59**
Positive for enterovirus (other sample types)				2
Positive for enterovirus from CSF				4
**Individuals screened for borreliosis/neuroborreliosis**				**131**
High IgG antibody level, indicating acute/recent borreliosis				20
Neuroborreliosis (high intrathecal index)				16
Suspected neuroborreliosis				1
**Individuals screened for TBEV infection**				**558**
Acute TBEV infection				28
**Other diagnosis listed in laboratory system**				**48**
Encephalitis without a known etiological agent				23
Acute infection without a known etiological agent				16
Autoimmune encephalitis				1
Acute disseminated encephalomyelitis				1
Demyelinating disease of the central nervous system				1
Meningitis				3
Myelitis				2
Fifth disease				1
Bacterial infection indicated in the laboratory system				
CSF				3
BSI				1
Sinusitis				1
Legionellosis				1
**Symptoms of the patients**				
Headache, neck pain and/or stiffness				19
Fever				11
Visual disturbance				6
Facial paralysis				3
Balance disorder				2
Loss of cognition				6
Tactile disturbance				2
Pain				4
Cramping				2
Motor weakness				2
Upper respiratory symptoms				1
Papilledema				1
Infertility				1
**SUMMARY**				
**All individuals**				**558**
**No causative agent or diagnosis listed in laboratory system**				**430 (430/558; 77%)**
**Causative agent detected (microbial)**				**80**
**Other diagnosis listed in laboratory system**				**48**

HSV, herpes simplex virus; VZV, varicella zoster virus; TBEV, tick-borne encephalitis virus; CSF, cerebrospinal fluid; BSI, bloodstream infection

**Table 2:** List of differential diagnostics and symptoms of the Kenyan patients (N = 60)

**Dengue virus diagnostics**	**Number of patients**
IgG positive, IFA	6
IgM positive, IFA	5
IgM positive, ELISA	1
At least one positive result from dengue virus diagnostics	7
**Chikungunya virus diagnostics**	
IgG positive, IFA	3
IgM positive, IFA	9
IgM positive, ELISA	6
**Sindbis virus diagnostics**	
IgG positive	19
IgM positive	0
**Symptoms of the patients**	
Rash	1
Joint pain	28
Myalgia	24
Vomiting	10
Diarrhea	6
Headache	5
Cough	9
Runny nose	9
Oral sores	2
Shivers	1
Odynophagia	1
Convulsions	1
Jaundice	1
Dysuria	2
Pharyngitis	1
Nausea	1

IFA, immunofluorescence assay; ELISA, enzyme-linked immunosorbent assay

All assays are described in more detail by Masika et al. [19]

RNA was extracted from human samples using a semi-automated MagNA Pure 96 System (Roche) and a Total Nucleic Acid Kit according to the manufacturer’s instructions. Extracted RNA samples were stored at −70°C.

### Bat samples

All bat samples were collected in Taita-Taveta County in southeastern Kenya between August 2021 and May 2023. Permission to conduct bat fieldwork was granted through permits from the National Commission for Science, Technology and Innovation (#NACOSTI/P/21/9267), the Kenya Wildlife Service (#KWS/BRM/500 and WRTI/RP/118.6), and the University of Arkansas Institutional Animal Care and Use Committee (#22012).

Bats were captured in flyways, building roosts, and inside roosting buildings, using mist-nets or by hand. Bats were placed into individual cotton bags upon capture and stored in a humid cool location overnight. The following morning, the species, sex, and age of the bats were recorded (Table 3). Fecal samples were collected from inside the bags and directly from the excreting bats during the sampling process. This capturing protocol followed previously described standards [23]. At dusk on the day after capture, the bats were returned to the collection site and released. The fecal pellets were stored in 2-ml tubes containing 0.5 ml of RNAlater, first at −20°C for a short initial period, and then at −80°C for long-term storage. Later, a subset of 340 samples was selected for RNA extraction using Invitrogen TRIzol Reagent (Thermo Fisher Scientific, Waltham, Massachusetts, USA) according to the manufacturer’s guidelines.

**Table 3:** Screened bat species, the number of samples screened (total N = 340) and the number of positive samples, age, and sex distribution of the individual bats

Bat species	n/positives
Little free-tailed bat (*Mops pumilus*)	136/0
Angolan free-tailed bat (*Mops condylurus*)	132/2
Heart-nosed bat (*Cardioderma cor*)	31/0
Andrew Rebori's house bat (*Scotophilus andrewreborii*)	13/0
Banana serotine (*Afronycteris nanus*)	6/0
Hinde's lesser house bat (*Scotoecus hindei*)	5/0
Egyptian fruit bat (*Rousettus aegyptiacus*)	4/0
Wahlberg's epauletted fruit bat (*Epomophorus wahlbergii*)	3/0
Sundevall’s roundleaf bat (*Hipposideros caffer*)	3/0
Yellow-winged bat (*Lavia frons*)	2/0
Silvered bat (*Glauconycteris argentata*)	1/0
Geoffroy's horseshoe bat (*Rhinolophus clivosus*)	1/0
Lander’s horseshoe bat (*Rhinolophus landeri*)	1/0
Egyptian free-tailed bat (*Tadarida aegyptiaca*)	1/0
African trident bat (*Triaenops afer*)	1/0
**Age and sex distribution of individual bats**	
**Age**	
Adult	343/2
Juvenile	24/0
**Sex**	
Female	208/0
Male	177/2

### Nested pan-PCR assay and screening

Previously designed PCR primers [20] were used to establish a nested pan-PCR protocol for detection of different members of the family *Paramyxoviridae*. In addition, for this assay, we designed a PCR control with an artificial built-in modification as a contamination control to avoid false positives (see Appendix). According to the taxonomy nomenclature at the time of publication, the viruses amplified with these PCR primers included members of the genera *Henipavirus*, *Morbillivirus*, *Respirovirus*, and *Rubulavirus*. Under the current taxonomy, the viruses detected using these primers would be classified in several genera of the two subfamilies *Orthoparamyxovirinae* and *Feraresvirinae*. Using these primers and various PMV controls, PCR products ranging in length from 600 to 700 base pairs were produced and then sequenced by the Sanger method. As the protocol was established for diagnostic purposes, it was further tested with human serum and CSF samples. To enhance the specificity of the assay for clinical samples, the level of nonspecific amplification of human DNA was reduced by using a touch-down PCR as the second PCR cycle, by first allowing flexible primer binding followed by more-specific amplification conditions towards the end of the procedure (see Appendix).

To test the performance of the nested pan-PCR assay, viral RNA from measles virus, canine morbillivirus (previously canine distemper virus, CDV), HeV G1, and NiV were used. Because HeV and NiV are classified as biosafety-level-4 pathogens, they were received as inactivated virus stocks from the Commonwealth Scientific and Industrial Research Organisation (CSIRO, Australia). In addition, we designed a cDNA plasmid as an assay contamination control. This plasmid contained part of the genome of CDV, with a recognizable insertion of artificial and non-coding nucleotides (CDV plasmid; Integrated DNA Technologies) to further discriminate possible contamination from the original viruses (Supplementary Fig. S1). To assess the sensitivity of the nested pan-PCR assay, a tenfold dilution series of CDV plasmid was tested, as well as RNAs from HeV G1 and NiV (Fig. 2).

All human and bat samples were screened using the nested pan-PCR assay, followed by agarose gel electrophoresis (AGE), sequencing and annotation using the core nucleotide collection from the National Centre for Biotechnology Information (NCBI) with Basic Local Alignment Tool (BLAST).

### Henipavirus RT-qPCR assays

Specific RT-qPCR assays for NiV and HeV G1 and G2 were established using the CFX96 platform (Bio-Rad) (see Appendix). Primers were designed to recognize the region of the genome encoding the nucleocapsid protein. All of the RT-qPCR assays were validated using viral RNA of NiV and HeV G1, commercial quantified plasmid controls of HeV G1 (IDT) and G2 (IDT), and a negative control panel (Table 4). The sensitivity was determined by calculating the limit of detection (LOD) and intra-assay repeatability for 10 parallel reactions.

**Table 4:** Cross-testing of henipavirus RT-qPCR assays

		Hendra-RT-qPCR (result: neg/pos)	Nipah-RT-qPCR (result: neg/pos)
**Virus RNA**	Canine distemper virus (in-house)	Neg	Neg
	Measles virus (vaccine strain)	Neg	Neg
	Yellow fever virus (17D, vaccine strain)	Neg	Neg
	Yellow fever virus (Asibi, vaccine strain)	Neg	Neg
	Zika virus (strain MR766)	Neg	Neg
	Dengue virus 1 (Zeptometrix)	Neg	Neg
	Dengue virus 3 (Zeptometrix)	Neg	Neg
	Reston ebolavirus (PHE)**	Neg	Neg
	Tai Forest virus (PHE)**	Neg	Neg
	Marburg virus (Musoke, PHE)**	Neg	Neg
	Bundibugyo virus (PHE)**	Neg	Neg
	Zaire ebolavirus (PHE)**	Neg	Neg
	Lassa virus (RKI)**	Neg	Neg
	Lymphocytic choriomeningitis virus (in-house)	Neg	Neg
	Crimean-Congo hemorrhagic fever virus (RKI)**	Neg	Neg
	Rift-Valley fever virus (RKI)**	Neg	Neg
	Parainfluenza 4 virus (in-house)	Neg	Neg
	Rinovirus 1B (in-house)	Neg	Neg
	Metapneumovirus (in-house)	Neg	Neg
	Respiratory syncytial virus (in-house)	Neg	Neg
	Hendra virus (CSIRO)	Pos	Neg
	Nipah virus (CSIRO)	Neg	Pos
**Plasmids**	Commercial plasmid-based construct: Nipah (IDT); 4.4E + 8 copies per reaction*	Neg	Pos
	Commercial plasmid-based construct: HeV G1 (IDT); 4.3E + 8 copies per reaction*	Pos	Neg
	Commercial plasmid-based construct: HeV G2 (IDT); 8.4E + 8 copies per reaction*	Pos	Neg
**Respiratory samples, altogether N = 9**	Rhinovirus positive, N = 6	Neg (6/6)	Neg (6/6)
	Rhino-, adeno- and parainfluenza virus 4 positive, N = 1	Neg (1/1)	Neg (1/1)
	Rhino- and enterovirus positive, N = 1	Neg (1/1)	Neg (1/1)
	Enterovirus positive, N = 1	Neg (1/1)	Neg (1/1)
Other sample types, negative***	Serum, N = 20	Neg (20/20)	Neg (20/20)
	CSF, N = 5	Neg (5/5)	Neg (5/5)
	EDTA-blood, N = 20	Neg (20/20)	Neg (20/20)

*Challenging the RT-qPCR with high load of control; Cycle values of 13.9 and 10.6 for commercial Nipah and Hendra plasmid controls, respectively. IDT, Integrated DNA Technologies; CSF, cerebrospinal fluid; CSIRO, Commonwealth Scientific and Industrial Research Organisation (Australia). **Viral RNAs kindly provided by the Public Health Institute (PHE, Porton Down, UK) and the Robert Koch Institute (RKI, Germany). *** Blood samples were originally from previously healthy individuals screened for human herpesvirus 6 nucleic acids.

## Results

### Nested pan-PCR testing of human samples

In total, 606 serum and 13 CSF samples from Finnish patients, as well as 60 serum samples from Kenyan patients (total N = 679) were screened using the nested pan-PCR assay. PCR products (~ 650 base pair long) were obtained from 16 sera (16/666, 2.4%) and two CSF samples (2/13, 15.4%), all of which were successfully sequenced. No viral nucleic acids were detected among the sequenced amplicons, and since all of these sequences corresponded to human DNA, they were interpreted as nonspecific background amplifications. No PMVs were detected in the Finnish and Kenyan patient samples.

### Nested pan-PCR testing of bat samples

**Fig. 1:**
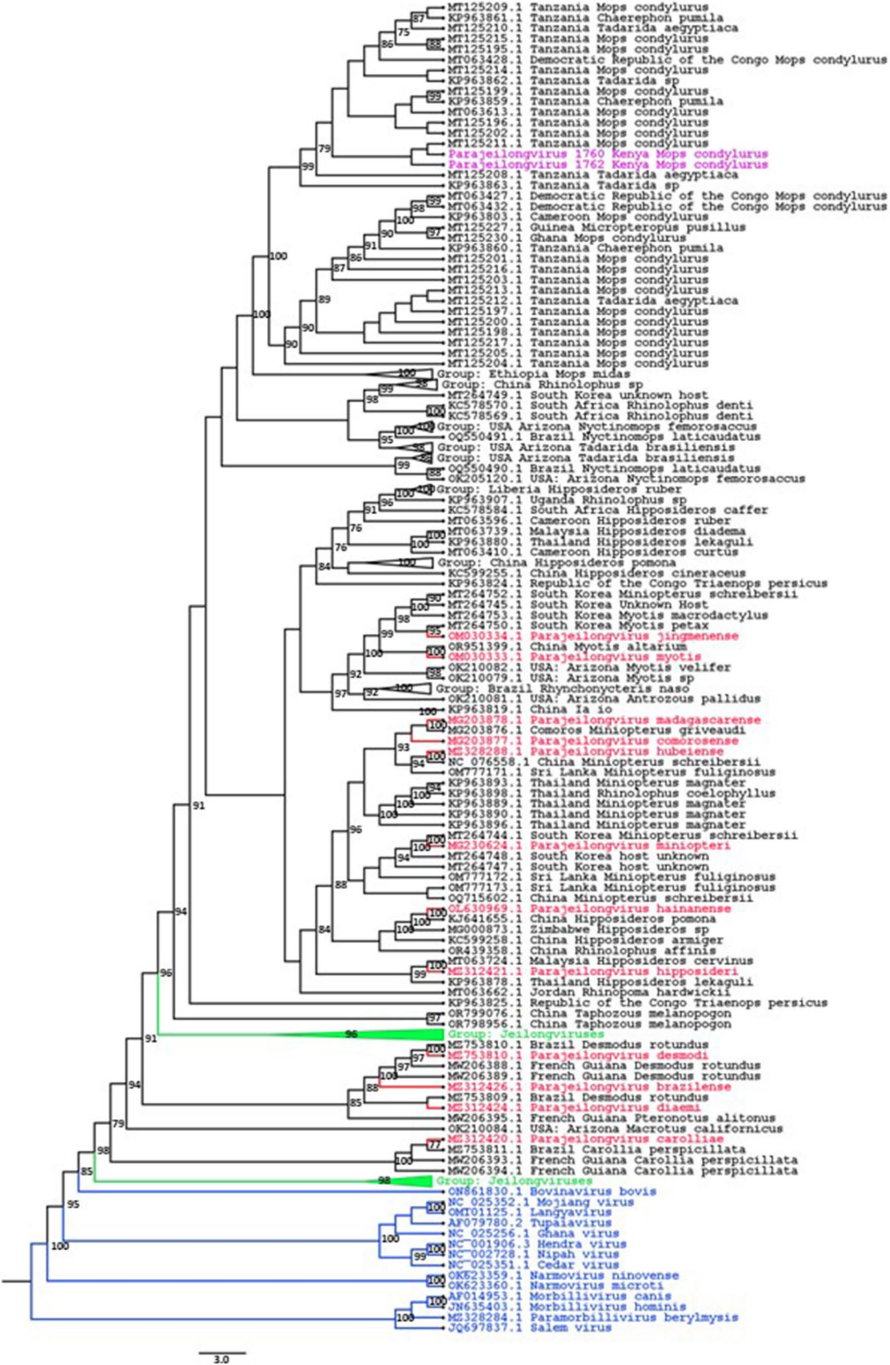
Maximum-likelihood phylogenetic tree based on partial sequences of L gene of the two detected parajeilongviruses and related viruses with midpoint rooting. The tree was constructed using sequences from orthoparamyxoviruses (blue), jeilongviruses (green), and parajeilongviruses (red), as well as the 100 top BLAST hits (black) for the two detected parajeilongviruses (purple). Strains found in the official ICTV taxonomy are labeled with their taxonomic nomenclature, whereas the sequences identified using BLAST are named using their geographical location and host species. The best-fitting model was chosen for these 475-base-pair-long sequences using IQTREE ModelFinder [35]. The analysis was performed in IQTREE [36], using the GTR + F + R6 model with 1000 bootstrap replicates, and the tree was visualized using FigTree v1.4.4 [26]. A full non-collapsed tree is shown in Supplementary Fig. S2)

In total, 340 bat fecal samples from 340 individuals comprising 16 different bat species (Table 3) from Taita-Taveta County (Kenya) were screened by nested pan-PCR, and amplification products of PMVs were detected in two fecal samples of Angolan free-tailed bats (*Mops condylurus*). The positive fecal samples were collected from two adult male bats in May 2023 from the main building of an orphanage house. The 457-base-pair-long sequences were first annotated using the BLAST annotation tool, followed by construction of a Bayesian maximum-likelihood phylogenetic tree using IQ-TREE [24, 25], which was visualized using FigTree [26] (Fig. 1 and Supplementary Fig. S2). This analysis revealed that these sequences shared a high degree of similarity with viral sequences from the genera *Jeilongvirus* and *Parajeilongvirus* of the subfamily *Orthoparamyxovirinae*. The highest sequence identity values – 99.11% and 98.66% – were observed with partial parajeilongvirus sequences obtained from *M. condylurus* samples from Tanzania. In addition, these sequences shared a high degree of similarity with partial viral sequences from free-tailed bats (*Tadarida* sp.). Unfortunately, no remaining sample material was available for further NGS sequencing attempts with these two positive bats.

**Fig. 2:**
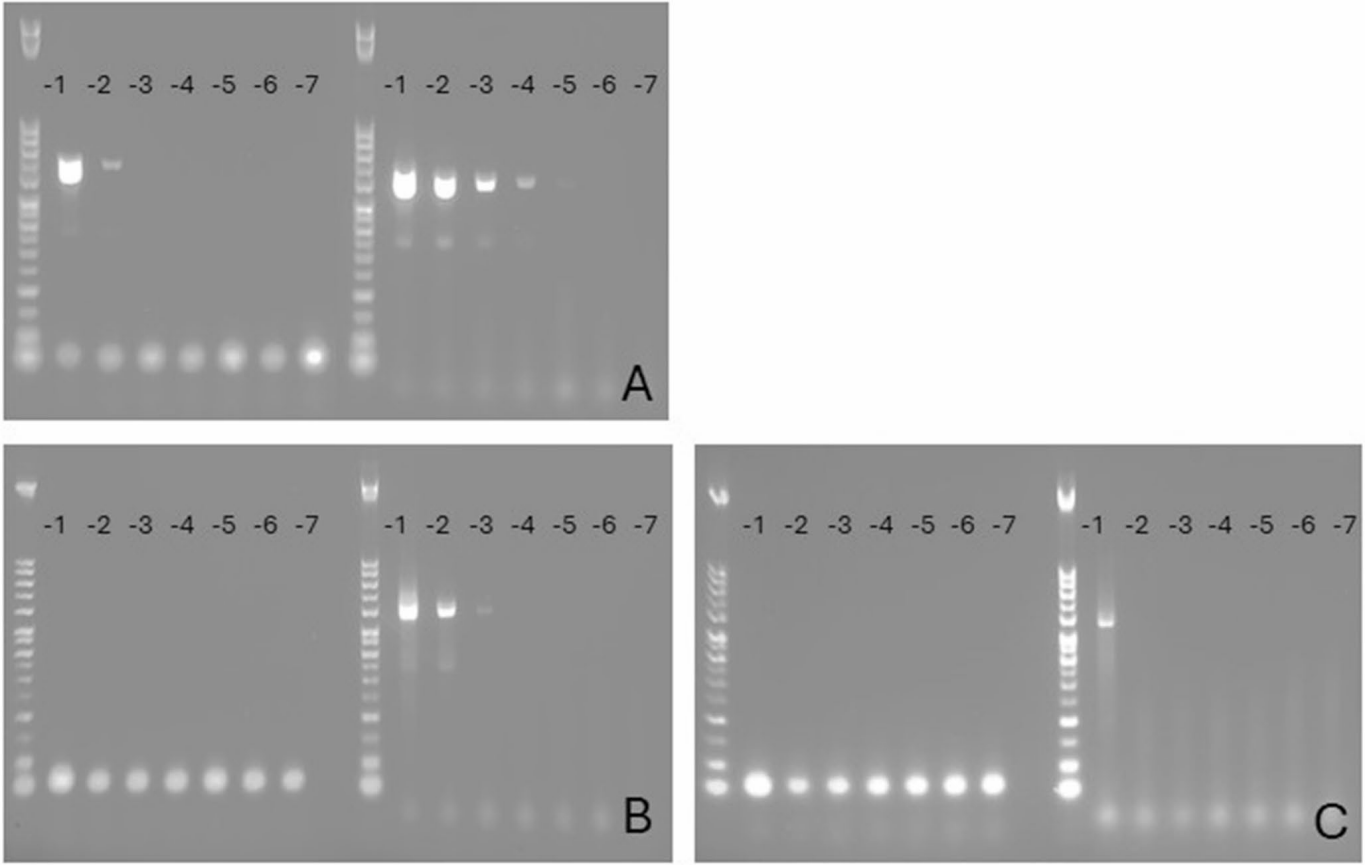
Dilution series of CDV plasmid and Nipah virus and Hendra virus RNAs using agarose gel electrophoresis (AGE). The performance between the first round of PCR followed by the nested round of PCR was compared using a tenfold dilution series of CDV plasmid control (IDT; panel A), as well as Nipah virus RNA (CSIRO; panel B) and Hendra virus RNA (CSIRO; panel C). The PCR products with a size of ~ 650 bp from the first and second PCR cycle are shown on the left and right, respectively. All PCR runs also included negative and CDV-positive controls (not shown in these AGE images). CSIRO, Commonwealth Scientific and Industrial Research Organisation (Australia)

Extracted RNA from CDV, measles virus, HeV, NiV, and a commercial plasmid control for CDV with a built-in contamination control were all successfully amplified using the nested pan-PCR and were subsequently sequenced and correctly annotated (see Appendix). The negative control and negative panel were all negative; no amplification was detected by AGE using these CSF or blood samples. Tenfold dilution series of CDV plasmid and HeV and NiV RNAs showed increased sensitivity with the nested pan-PCR assay compared to the initial PCR step of the assay (Fig. 2). The CDV control demonstrated clear amplification already after the first PCR step, whereas HeV and NiV could be detected only after the nested step. The nested PCR step increased performance by 1000-fold with the CDV plasmid control (Fig. 2).

### Henipavirus RT-qPCR assays

The assays were validated using extracted viral RNA from inactivated HeV and NiV (CSIRO) and commercial quantified plasmid controls of NiV, HeV G1, and HeV G2 (IDT). The henipavirus RT-qPCR assays were able to discriminate NiV from HeV. A negative panel comprised of 14 sera, five cerebrospinal fluid samples, 20 EDTA-blood samples, and nine respiratory samples, in addition to a panel of 20 non-henipaviral RNAs (Table 4), were all negative (68/68, 100%). By using 95% confidence intervals (SPSS, Probit, IBM), the LOD for the henipavirus RT-qPCRs was 14.1, 7.2, and 9.8 copies per PCR reaction for HeV G1 (IDT), HeV G2 (IDT), and NiV (IDT), respectively. At a copy level of 430–440 with 10 parallel reactions for HeV G1, the average Ct value was 33.4, the standard deviation (STDEV) was 1.1, and the coefficient of variation (CV) was 3%. At a copy level of 8400 for HeV G2, the Ct value was 35.5, the STDEV was 0.78, and the CV was 2%, and for NiV at a copy level of 430–440, the Ct value was 32.5, the STDEV was 0.70, and the CV was 2%.

## Discussion

In Finland, no endemic zoonotic infections caused by PMVs have been reported to date. However, another PMV – measles virus – is still causing sporadic infections almost annually. The highest rate of measles virus infections was in 2011, with 27 laboratory-confirmed cases [27]. The coverage of measles, mumps, and rubella (MMR) vaccination varies geographically in Finland but is high overall [27]. In this study, as expected, we did not find measles virus or other PMVs in the Finnish samples (N = 619). The Kenyan patient samples were collected from April to August 2016, before the large-scale measles-rubella vaccination campaign started in October 2016 in Kenya. Nevertheless, we did not find any measles virus or other PMVs in the Kenyan samples. However, it is worth noting that we did not have any vaccination data from these 60 Kenyan patients. Although measles vaccines have been used for decades in Kenya, measles virus continues to cause infections every year. For example, in 2016, the vaccination coverage for the first and second dose of the measles vaccine was 93% and 35%, respectively. In the same year, 128 measles cases were reported in Kenya[28]. Of note, there is currently a measles epidemic ongoing in the USA [29].

**Fig. 3:**
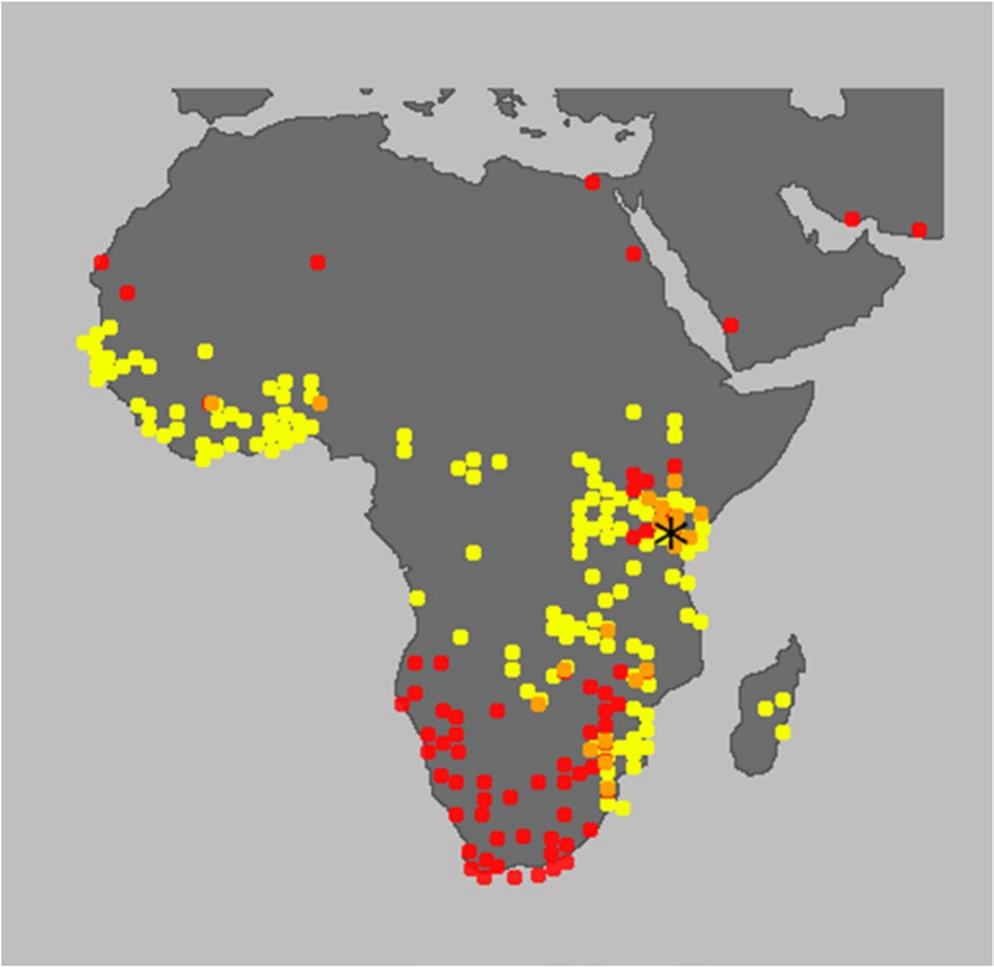
Map highlighting the geographical distribution of *Mops condylurus* (yellow) and *Tadarida aegyptia* (red) and their region of overlap (orange). The sample collection site is indicated by an asterisk [32, 33]

Complete genome sequences have been obtained for paramyxoviruses from bat samples collected in Kenya, including samples from *Triaenops*, *Hipposideros*, *Coleura*, *Miniopterus*, *Otomops*, *Nycteris*, *Rousettus*, *Cardioderma* and *Chaerephon* sp., but none have been obtained from *Mops* species [30, 31]. In our study, a parajeilongvirus was detected in two individual *Mops condylurus* fecal samples, using nested pan-PCR and sequencing. The sequences from these bat samples exhibited high sequence similarity (90–99% identity) to other partial genome sequences of putative paramyxoviruses found in different bat species, i.e., *Mops condylurus, Mops pumilus*, and *Tadarida* sp., in northern Africa (Fig. 1). Partial genome sequences of parajeilongviruses found in Tanzania, obtained from the NCBI GenBank database, were 98–99% identical to our sequences, suggesting the circulation of this parajeilongvirus type in the African Great Lakes region. The similarity of the viral sequences in *Mops* sp. and closely related *Tadarida* sp. suggests potential for spillover between these species. However, due to the lack of whole genome sequences, and because only a highly conserved part of the sequence was used to study the relationships among different virus strains, no definitive conclusions are possible. Although *Tadarida aegyptiaca* is generally found in more-southern parts of Africa than *M. condylurus*, they are both present in the African Great Lakes region, especially in Kenya (Fig. 3) [32, 33].

Taxonomically, the genus *Parajeilongvirus* was separated from the genus *Jeilongvirus* in 2023 based on differences in their host associations and genomic structure (International Committee on Taxonomy of Viruses). In the current taxonomy, all of the parajeilongviruses originate mostly from bats, which is consistent with our findings, since the sequences obtained from *M. condylurus* samples were more similar to parajeilongviruses than jeilongviruses (Fig. 1). Both genera consist of newly discovered viruses, and therefore, in-depth research data on these viruses is limited. Although new viruses are detected continually, the most common screening methods in use result in short nucleotide sequences from conserved areas of the genome, leaving many open questions, including the detailed characteristics of the detected viruses.

Overall, the procedure for the nested pan-PCR assay fits well into a routine diagnostic setting, and in this study, no cross-contamination was detected. The nested PCR step includes a contamination control that appears to be suitable for routine diagnostic purposes. The assay produced some background amplification (human DNA), more frequently with CSF samples (15.4%) than with serum samples (2.4%) , but this was technically straightforward to exclude by Sanger sequencing. The process of implementing a plasmid control improved the practicality of the laborious contamination-prone nested PCR in routine diagnostics, highlighting its potential application, for example, in resource-limited settings where RT-qPCR or next-generation sequencing may not be available. More nonspecific amplification was detected by AGE when using samples from bats than when using human samples. This was expected, as bat fecal samples typically include a greater diversity of background nucleic acids than human blood or CSF samples.

For emergency settings, molecular methods based on nested PCR and sequencing are not recommended due to their time-consuming nature and susceptibility to cross-contamination. For situations in which results are needed within hours rather than days, RT-qPCR assays with a short turnaround time and easily interpreted results should be designed. In this study, we designed, performed, and validated specific RT-qPCR assays for NiV, HeV G1, and HeV G2. These assays demonstrated good performance, including efficient detection of the new HeV G2. These methods are now available for use in clinical diagnostics. Although RT-qPCR assays are efficient, conventional PCR assays combined with Sanger sequencing remain valuable tools for distinguishing new virus variants and genotypes from existing ones [21].

Preparedness for zoonotic infections requires basic research and surveillance to enhance our understanding of viral circulation in humans and wildlife. The family *Paramyxoviridae* is likely to include numerous undiscovered zoonotic pathogens. A recent example of a spillover transmission from animals to humans is the shrew-borne Langya virus, which infected 35 people in China in 2022 [34]. Due to global travel and constant human interactions, pandemic risk assessment must extend beyond national borders. Rapid and efficient clinical tools, such as qPCR, for diagnosing travel-associated infections, combined with the surveillance of local zoonotic risks in wildlife, are essential for preventing emerging virus infections and viral transmission. The paramyxovirus surveillance methods described here were used for screening both human and bat samples and could be coupled with full-genome sequencing when possible. In the future, these methods can be applied to other wildlife samples, including those from rodents and shrews, to further uncover the diversity of paramyxoviruses and to assess potential local zoonotic risks in Finland, Kenya, and elsewhere.

## Electronic Supplementary Material

Below is the link to the electronic supplementary material

Supplementary File 1 (DOCX 778 KB)

## Abbreviations

AGE: agarose gel electrophoresis
BLAST: Basic Local Alignment Search Tool
CDV,: canine distemper virus
CNS: central nervous system
CSF: cerebrospinal fluid
CSIRO: Commonwealth Scientific and Industrial Research Organisation
CV: coefficient of variation
HUS: University Hospital of Helsinki
HeV: Hendra virus
HeV G1: Hendra virus genotype 1
HeV G2: Hendra virus genotype 2
IDT: Integrated DNA Technologies
LOD: limit of detection
MMR: measles, mumps and rubella vaccine
NCBI: National Center for Biotechnology Information
NiV: Nipah virus
PMV: paramyxovirus
RT-PCR: reverse transcription polymerase chain reaction
STDEV: standard deviation

## References

[1] Jones KE et al (2008) Global trends in emerging infectious diseases. Nat 451(7181):990–993 10.1038/nature06536 10.1038/nature06536 PMC5960580 18288193

[2] World Health Organization (WHO) https://www.who.int/health-topics/measles#tab=tab_1.

[3] Kitchen A, Shackelton LA, Holmes EC (2011) Family level phylogenies reveal modes of macroevolution in RNA viruses. Proc Natl Acad Sci U S A 108(1):238–243. 10.1073/PNAS.1011090108 10.1073/pnas.1011090108 PMC3017157 21173251

[4] Mahalingam S et al (2012) Hendra virus: an emerging paramyxovirus in Australia. Lancet Infect Dis 12(10):799–807. 10.1016/S1473-3099(12)70158-5 10.1016/S1473-3099(12)70158-5 22921953

[5] Eaton BT, Broder CC, Middleton D, Wang LF (2006) Hendra and Nipah viruses: different and dangerous. Nat Rev Microbiol 4(1)23–35. 10.1038/NRMICRO1323 10.1038/nrmicro1323 PMC7097447 16357858

[6] Halpin K et al (2011) Pteropid bats are confirmed as the reservoir hosts of henipaviruses: a comprehensive experimental study of virus transmission. Am J Trop Med Hyg 85(5):946–951. 10.4269/AJTMH.2011.10-0567 10.4269/ajtmh.2011.10-0567 PMC3205647 22049055

[7] Islam MS et al (2016) Nipah Virus Transmission from Bats to Humans Associated with Drinking Traditional Liquor Made from Date Palm Sap, Bangladesh, 2011–2014. Emerg Infect Dis 22(4):664–670. 10.3201/EID2204.151747 10.3201/eid2204.151747 PMC4806957 26981928

[8] Thiagarajan K (2023) Nipah virus: India’s Kerala state moves quickly to control fresh outbreak. BMJ 382. 10.1136/BMJ.P2117 10.1136/bmj.p2117 37714528

[9] Drexler JF et al (2012) Bats host major mammalian paramyxoviruses. Nat Commun 3. 10.1038/NCOMMS1796 10.1038/ncomms1796 PMC3343228 22531181

[10] Maganga GD et al (2014) Identification of an unclassified paramyxovirus in Coleura afra: a potential case of host specificity. PLoS ONE 9(12). 10.1371/JOURNAL.PONE.0115588 10.1371/journal.pone.0115588 PMC4281239 25551455

[11] Felix Drexler J et al Henipavirus RNA in African Bats. 10.1371/journal.pone.0006367

[12] Weiss S et al (2012) Henipavirus-related Sequences in Fruit Bat Bushmeat, Republic of Congo - 18, Number 9—September 2012 - Emerging Infectious Diseases journal - CDC. Emerg Infect Dis 18(9):1536–1537. 10.3201/EID1809.111607 22935105 10.3201/eid1809.111607 PMC3437727

[13] Atherstone C et al (2019) Evidence of exposure to henipaviruses in domestic pigs in Uganda Funding information CGIAR Research Program on Agriculture for Nutrition and Health. Transbound Emerg Dis 66. 10.1111/tbed.13105 10.1111/tbed.13105 PMC6849855 30576076

[14] Albariño CG et al (2014) Novel Paramyxovirus Associated with Severe Acute Febrile Disease, South Sudan and Uganda, 2012, Emerg Infect Dis 20(2):211 10.3201/EID2002.131620 10.3201/eid2002.131620 PMC3901491 24447466

[15] Kurth A et al (2012) Novel paramyxoviruses in free-ranging European bats. PLoS ONE 7(6). 10.1371/journal.pone.0038688 10.1371/journal.pone.0038688 PMC3380927 22737217

[16] Haring VC et al (2024) Detection of novel orthoparamyxoviruses, orthonairoviruses and an orthohepevirus in European white-toothed shrews DATA SUMMARY. Microb Genom 10:1275. 10.1099/mgen.0.001275 10.1099/mgen.0.001275 PMC11293873 39088249

[17] Horemans M, Van Bets J, Maes J, Maes P, Vanmechelen B (2023) Discovery and genome characterization of six new orthoparamyxoviruses in small Belgian mammals. Virus Evol. 10.1093/ve/vead065 38034864 10.1093/ve/vead065 PMC10684267

[18] Vanmechelen B, Vergote V, Merino M, Verbeken E, Maes P (2020) Common occurrence of Belerina virus, a novel paramyxovirus found in Belgian hedgehogs. Sci Rep 10(1). 10.1038/S41598-020-76419-1 10.1038/s41598-020-76419-1 PMC7653956 33168902

[19] Kyheröinen) E-MT, Liukko U-M, Stjernberg T (2019) Atlas of Finnish Bats, Ann Zool Fennici 56(1–6):207–250. 10.5735/086.056.0117

[20] Tong S, Chern SWW, Li Y, Pallansch MA, Anderson LJ (2008) Sensitive and broadly reactive reverse transcription-PCR assays to detect novel paramyxoviruses. J Clin Microbiol 46 (8):2652–2658. 10.1128/JCM.00192-08 10.1128/JCM.00192-08 PMC2519498 18579717

[21] Annand EJ et al (2022) Novel Hendra Virus Variant Detected by Sentinel Surveillance of Horses in Australia. Emerg Infect Dis 28(3):693–704. 10.3201/EID2803.211245 10.3201/eid2803.211245 PMC8888208 35202527

[22] Masika MM et al (2020) Detection of dengue virus type 2 of Indian origin in acute febrile patients in rural Kenya. PLoS Negl Trop Dis 14(3):e0008099. 10.1371/JOURNAL.PNTD.0008099 10.1371/journal.pntd.0008099 PMC7069648 32126086

[23] Sikes RS (2016) 2016 Guidelines of the American Society of Mammalogists for the use of wild mammals in research and education. J Mammal 97(3):663–688. 10.1093/JMAMMAL/GYW078 10.1093/jmammal/gyw078 PMC5909806 29692469

[24] Kalyaanamoorthy S, Minh BQ, Wong TKF, Von Haeseler A, Jermiin LS (2017) ModelFinder: Fast Model Selection for Accurate Phylogenetic Estimates. Nat Methods 14(6):587. 10.1038/NMETH.4285 10.1038/nmeth.4285 PMC5453245 28481363

[25] Minh BQ et al (2020) IQ-TREE 2: New Models and Efficient Methods for Phylogenetic Inference in the Genomic Era. Mol Biol Evol 37(5):1530–1534. 10.1093/MOLBEV/MSAA015 10.1093/molbev/msaa015 PMC7182206 32011700

[26] Rambaut A (2018) FigTree ver 1.4.4. Institute of Evolutionary Biology. University of Edinburgh, Edinburgh

[27] Finnish Institute for Health and Welfare Finnish National Infectious Diseases Register. https://thl.fi/aiheet/infektiotaudit-ja-rokotukset/seurantajarjestelmat-ja-rekisterit/tartuntatautirekisteri/tartuntatautien-esiintyvyystilastot/tartuntatautien-esiintyvyys-suomessa-raportit

[28] World Health Organization Estimates of National Immunization Coverage

[29] U.S Centres for Disease Control and Prevention

[30] Conrardy C et al (2014) Molecular Detection of Adenoviruses, Rhabdoviruses, and Paramyxoviruses in Bats from Kenya. Am J Trop Med Hyg 91(2):258–266. 10.4269/AJTMH.13-0664 10.4269/ajtmh.13-0664 PMC4125246 24865685

[31] Waruhiu C et al (2017) Molecular detection of viruses in Kenyan bats and discovery of novel astroviruses, caliciviruses and rotaviruses. Virol Sin 32(2):101–114. 10.1007/S12250-016-3930-2 10.1007/s12250-016-3930-2 PMC6702250 28393313

[32] Taxonomy GBIFB (2023) Mops condylurus (A.Smith, 1833) in GBIF Secretariat. 10.15468/39omei. Checklist dataset

[33] Taxonomy GBIFB (2023) Tadarida aegyptiaca (E.Geoffroy, 1818) in GBIF Secretariat. 10.15468/39omei. Checklist dataset

[34] Zhang X-A et al (2022) A Zoonotic Henipavirus in Febrile Patients in China. N Engl J Med 387(5):470–472. 10.1056/NEJMC2202705 10.1056/NEJMc2202705 35921459

[35] Kalyaanamoorthy S, Minh BQ, Wong TKF, Von Haeseler A, Jermiin LS (2017) ModelFinder: Fast Model Selection for Accurate Phylogenetic Estimates. Nat Methods 14(6):587. 10.1038/NMETH.4285 10.1038/nmeth.4285 PMC5453245 28481363

[36] Minh BQ et al (2020) IQ-TREE 2: New Models and Efficient Methods for Phylogenetic Inference in the Genomic Era. Mol Biol Evol 37(5):1530–1534. 10.1093/MOLBEV/MSAA015 10.1093/molbev/msaa015 PMC7182206 32011700

