# Supplementary material for: New parajeilongviruses detected in bats but not in humans: assays for screening and diagnostic purposes
Source: Arch Virol. 2026 Jan 15;171(2):55. doi: 10.1007/s00705-025-06520-1 (PMC12808154; doi:10.1007/s00705-025-06520-1)
Supplement: Supplementary file 1 — (DOCX 778 KB) [file 705_2025_6520_MOESM1_ESM.docx]

New parajeilongviruses detected in bats but not in humans – assays for screening and diagnostic purposes

Emilia Pulkkinen MSc, Reilly Jackson PhD, Ruut Joensuu PhD, Essi M. Korhonen PhD, Moses Muia Masika PhD, Omu Anzala PhD, Joseph G Ogola PhD, Paul W Webala PhD, Tamika Joyce Lunn Assistant Professor, Kristian M. Forbes Associate Prof, Olli Vapalahti Prof, Tuure Kinnunen Prof, Tarja Sironen Prof^1^, Anne J. Jääskeläinen Doc^1^

**Appendix**

**Materials and methods**

**Nested-pan-PCR assay**

A nested-pan-PCR assay was performed using primers PAR-F1, PAR-F2 and PAR-R (Table Appendix 2), previously introduced by Tong et al [20]*.* These primers amplify a fragment of conserved area from the L-gene encoding RNA-dependent RNA-polymerase, yielding an amplicon size of 600–700 base pairs, depending on the target virus [Figure Appendix 1; plasmid control of CDV sequence with target primers (PCR product: 663 base pairs), and modification used to detect PCR contaminations].

The first PCR reaction was conducted using the Invitrogen SuperScript^TM^ III One-Step RT-PCR with Platinum^TM^ Taq PCR-kit (Thermo Fischer Scientific). The original concentrations of the PAR-F1 and PAR-R primers were titrated from 920 nm to 700 nm for optimal sensitivity for our PCR protocol. The optimized PCR reaction mix consisted of a final concentration of 1x master mix, 0.05 mM of MgSO_4_, 700 nm of PAR-F1 and PAR-R primers each, 0.80 µl enzyme mix and 7µl of RNA template. Water was added to reach a final volume of 25 µl. The RT-PCR reaction was sequentially incubated at 50°C for 30 min for the reverse transcriptase, 94°C for 2 min for the enzyme activation, followed by 45 cycles of 94°C for 20 sec, 50°C for 30 sec and 72°C for 60 sec, with a final extension at 72°C for 5 min.

The nested round of the PCR reaction was conducted using AllTaq Master Mix Kit (Qiagen) and PAR-F2 and PAR-R primers. The original concentration of the reverse primer PAR-R was titrated from 1 µM to 0.75 µM. The PCR reaction consisted of 1 unit of Alltaq Master mix, 1 µM PAR-F2, 0.75 µM PAR-R, 1 µl of the first PCR reaction product and water to reach a final volume of 25 µl. For initiation, the PCR reaction was heated to 95°C for 2 min to activate the DNA polymerase. The PCR started by denaturation at 94°C for 15 sec, annealing at 61°C for 30 sec and extension at 72°C for 30 sec, for a total of six times. The touch-down protocol was subsequently performed with 21 cycles of denaturation (94°C 15 sec), annealing (30 sec, temperature decreasing in steps) and extension (72°C 30 sec). The annealing temperature decreased by 0.5°C with every cycle, beginning at 61°C and ending at 51°C for the last cycle. After the touch-down cycles, continual annealing cycles at 94°C for 15 sec, 50°C for 30 sec and 72°C for 30 sec were repeated 16 times. The final extension was executed at 72°C for 5 min.

Controls (Table Appendix 1): To optimize the method, we used a dilution series of modified CDV plasmid (Integrated DNA Technologies, IDT; Figure Appendix 1), as well as viral RNAs of measles morbillivirus (University of Helsinki), CDV (University of Helsinki), and Hendra and Nipah virus RNA (CSIRO, Australia). The method was also tested to be adequate with parainfluenza virus 1 (HUS Diagnostic Center Helsinki). All positive control RNA templates were extracted from inactivated virus stocks, except for the parainfluenza virus 1 sample, which was an extracted clinical sample collected from the upper respiratory tract. For PCR screening of the clinical samples, only CDV was used as a positive control to trace possible contamination.

The performance between the first PCR step and the following nested round was evaluated with the dilution series of CDV plasmid control (IDT) and extracted Hendra and Nipah viral RNAs.

Examining the PCR amplicons: PCR products were run in a 2% agarose gel supplemented with GelRed Nucleic Acid Stain (Merck) and visualized with UV light. DirectLoad^TM^ 50 bp DNA Step Ladder (Merck) was run in parallel on the gels to estimate the amplicon size of the PCR products.

Sequencing: PCR products that showed amplification on agarose gels were individually selected for sequencing. The PCR amplicons of the positive control RNAs were also sequenced to control the sequencing protocol. Sanger sequencing was performed at BIDGEN (DNA Sequencing and Genomics, Institute of Biotechnology, University of Helsinki).

Sequence analysis and annotation: Sequences were analyzed using NCBI Basic Local Alignment Search Tool and nucleotide collection from NCBI (https://blast.ncbi.nlm.nih.gov/Blast.cgi).

**Henipavirus RT-qPCR assays**

Primers and probes for both Hendra and Nipah virus-specific RT-qPCR assays were designed using the Primer3-software [37]. The primers Nipah-FW and Nipah-RV amplify a product of 172 base pairs from the nucleocapsid protein region of the Nipah virus genome, which is detected by the probes Nipah1 and Nipah2 (Table Appendix 2). The primers Hendra-F, Hendra-F2, Hendra-R, and Hendra-R2 amplify a product of 222 base pairs from the nucleocapsid protein region of the Hendra virus genome, which is detected by the Hendra-Probe (Table Appendix 2).

The RT-qPCR assays for the Nipah virus and Hendra virus were carried out using an Invitrogen SuperScript^TM^ III One-Step RT-qPCR with Platinum^TM^ Taq PCR kit (Thermo Fischer Scientific). Primers and probes had the following final volumes: 600 nM Nipah-F and 600 nM Nipah-RV primers, 300 nM Nipah1-probe and 300 nM Nipah2-probe, and 400 nM all of Hendra primers and 400 nM Hendra-probe. In addition, 1x mastermix with 0.05 µl ROX, 7 µl of RNA template, and 0.5 µl RT enzyme were added. PCR-clean water was added to reach the final volume of 25 µl.

Both PCR reactions were carried out at 50°C for 30 min, followed by denaturation at 95°C for 2 min, and 45 cycles of amplification at 95°C for 15 s and 60°C for 50 s. Real-time detection was performed with CFX96 Touch Real-Time PCR Detection System (Bio-Rad, Finland).

Controls (Table Appendix 1): For setting up and optimizing the method, we used a dilution series of extracted RNAs Hendra virus (strain 024V-04685), and Nipah virus (strain 024V-04684), and plasmid controls of Hendra genotype 1 (IDT; NCBI GenBank: AF017149.3), Hendra genotype 2 (IDT; NCBI GenBank: MZ229748.1) and Nipah (IDT; NCBI GenBank: MK673563.1). These plasmids were constructed using part of the N-gene sequences of these viruses. All the positive control RNA templates were extracted from inactivated virus stocks. Henipaviruses are classified as biosafety level 4 pathogens, and were therefore received as inactivated virus stocks from CSIRO (Australia).

**Phylogenetic tree**

The partial sequences from Sanger sequencing were annotated against known paramyxovirus sequences using Basic Local Alignment Tool (BLAST, NCBI, USA). Sequences (457 base pairs in length) of the 100 best BLAST results and known jeilongviruses, parajeilongvisues and other orthoparamyxoviruses were aligned with CLUSTALW [38]. A Bayesian phylogenetic tree was configured using the maximum-likelihood tree structure based on a GTR+F+R6 model with 1000 bootstrap replicates in IQ-TREE [35,36]. The visualization was performed with FigTreev1.4.4. [26] with midpoint rooting.

Table Appendix 1. List of controls used for optimization, validation, and screening.

| **Control** | **GenBank/Strain** | **RNA/cDNA** | **Source** | **Implementation** |
| --- | --- | --- | --- | --- |
| Measles morbillivirus | vaccine strain | RNA | University of Helsinki, Finland | pan-PCR set-up |
| Canine morbillivirus (CDV) | In-house | RNA | University of Helsinki, Finland | pan-PCR set-up |
| CDV plasmid | KJ466106.1, modified | cDNA | IDT | pan-PCR set-up and screening control |
| Hendra virus | 024V-04685 | RNA | CSIRO, Australia | pan-PCR set-up, Henipavirus RT-qPCR validation |
| Hendra plasmid | AF017149.3 | cDNA | IDT | Henipavirus RT-qPCR validation and screening control |
| Hendra genotype 2 | MZ229748.1 | cDNA | IDT | Henipavirus RT-qPCR validation |
| Nipah virus | 024V-04684 | RNA | CSIRO, Australia | pan-PCR set-up, Henipavirus RT-qPCR validation |
| Nipah plasmid | MK673563.1 | cDNA | IDT | Henipavirus qPCR validation and screening control |

Table Appendix 2. List of primers and probes used in assays in this study.

| **Primer / probe** | **Sequence** | **Implementation** |
| --- | --- | --- |
| PAR-F1 | 5’-GAAGGNTATTGTCANAARNTNTGGAC-3’ | Nested pan-PCR |
| PAR-F2 | 5’ - GTTGCTTCAATGGTTCARGGNGAYAA -3' | Nested pan-PCR |
| PAR-R | 5’-GCTGAAGTTACNGGNTCNCCDATRTTNC –3' | Nested pan-PCR |
| Nipah-FW | 5’- GCAGGAAGGCAAGAGAGTAA -3’ | NiV RT-qPCR |
| Nipah-RV | 5’- ACACTGTYAGCAAGGGATGA -3’ | NiV RT-qPCR |
| Nipah1 | VIC-5’- AGGTGTGCTCATYGGAG -3’-MGBNFQ | NiV RT-qPCR |
| Nipah2 | VIC-5’-AGTGGCMGACAGTCAGTT -3’-MGBNFQ | NiV RT-qPCR |
| Hendra-F | 5’-GCTGGCGGGATYGAYCAARA-3’ | HeV RT-qPCR |
| Hendra-F2 | 5’- GCTGGCGGGATYGAYCAGAA -3’ | HeV RT-qPCR |
| Hendra-R | 5’-CTCACTGAYTKCCTACCACT-3’ | HeV RT-qPCR |
| Hendra-R2 | 5’- GTSACWGATTGCCTWCCTCT -3’ | HeV RT-qPCR |
| Hendra-Probe | 6-FAM-5’-AACTGGCTGCTGCAGTTC -3’-MGBNFQ | HeV RT-qPCR |

Figure Appendix 1. FASTA sequence used in the canine distemper virus (CDV) positive control plasmid. The plasmid was reconstructed using the genome of CDV (NCBI, KJ466106.1). The sequence modification (addition of non-coding nucleotides) and the predicted primer locations are bolded (PCR product size: 663 base pairs).


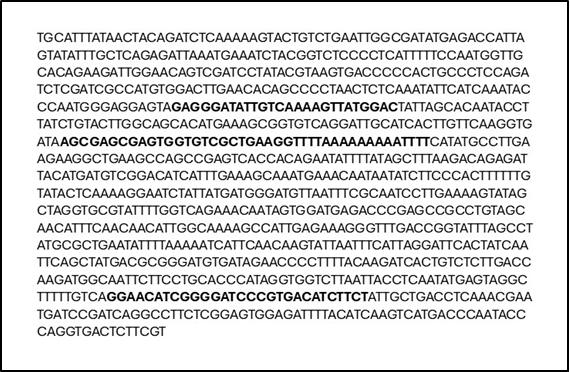


Figure Appendix 2. The original phylogenetic tree showing all the sequences used. Taxonomical names of jeilongviruses and parajeilongviruses derived from the ICTV taxonomy are indicated with green and red, respectively. Other orthoparamyxoviruses are indicated with blue. The results from BLAST annotation (black) are named with the geographical location and host species. The two detected partial genomes of parajeilongviruses are purple. The maximum-likelihood tree was constructed by using IQTREE [35,36] with the GTR+F+R6 model and 1000 bootstrap replicates with 457-base-pair-long sequences, and visualized with FigTree [26].


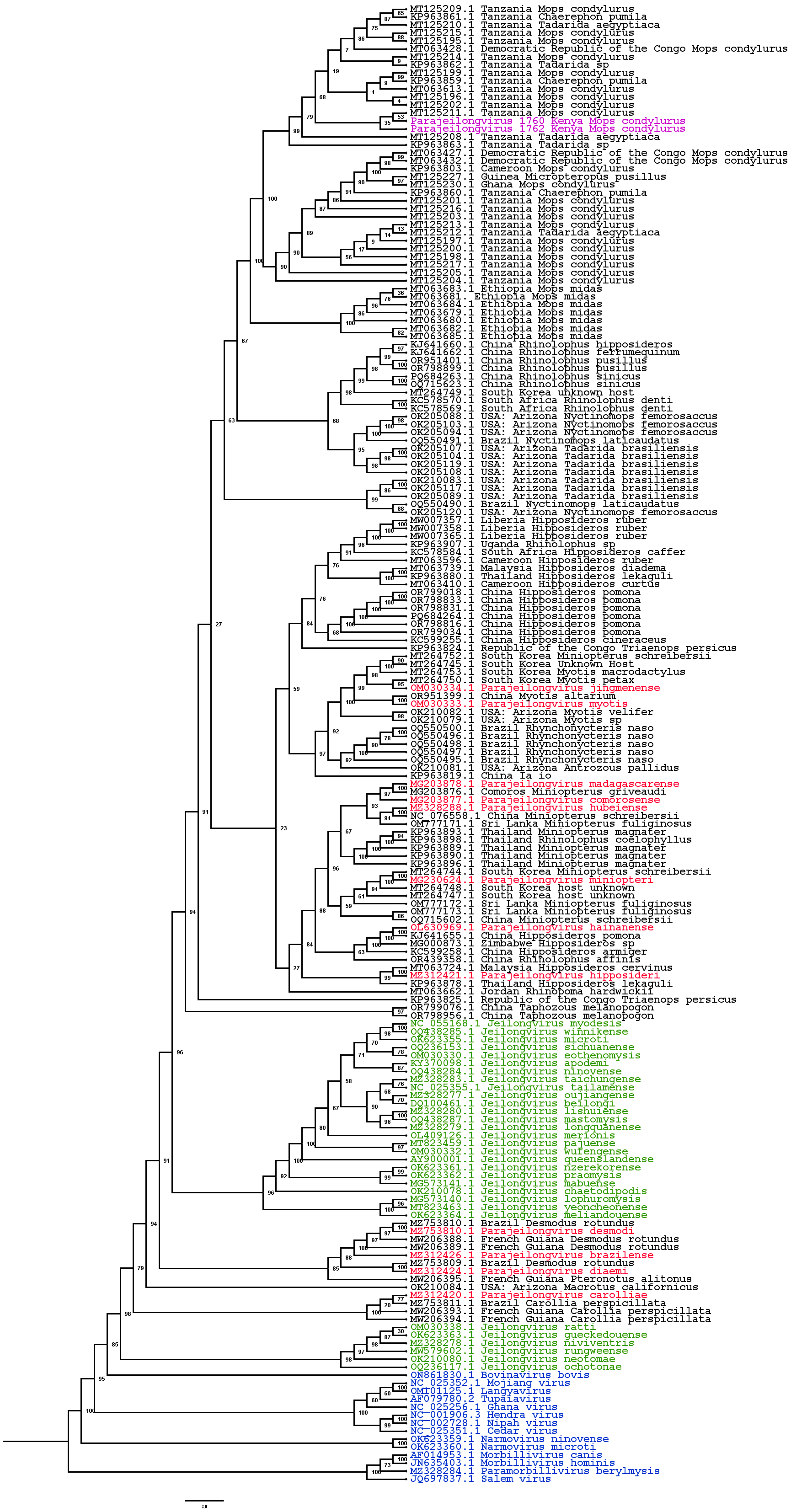


References:

[20] S. Tong, S. W. W. Chern, Y. Li, M. A. Pallansch, and L. J. Anderson, “Sensitive and broadly reactive reverse transcription-PCR assays to detect novel paramyxoviruses,” *J Clin Microbiol*, vol. 46, no. 8, pp. 2652–2658, Aug. 2008, doi: 10.1128/JCM.00192-08.

[26] A. Rambaut, “FigTree ver 1.4.4. Institute of Evolutionary Biology, University of Edinburgh, Edinburgh,” 2018.

[35] S. Kalyaanamoorthy, B. Q. Minh, T. K. F. Wong, A. Von Haeseler, and L. S. Jermiin, “ModelFinder: Fast Model Selection for Accurate Phylogenetic Estimates,” Nat Methods, vol. 14, no. 6, p. 587, May 2017, doi: 10.1038/NMETH.4285.

[36] B. Q. Minh et al., “IQ-TREE 2: New Models and Efficient Methods for Phylogenetic Inference in the Genomic Era,” Mol Biol Evol, vol. 37, no. 5, pp. 1530–1534, May 2020, doi: 10.1093/MOLBEV/MSAA015.

[37] Untergasser A, Cutcutache I, Koressaar T, Ye J, Faircloth BC, Remm M, Rozen SG. Primer3--new capabilities and interfaces. Nucleic Acids Res. 2012 Aug;40(15):e115. doi: 10.1093/nar/gks596. Epub 2012 Jun 22. PMID: 22730293; PMCID: PMC3424584.

[38] Larkin MA, Blackshields G, Brown NP, Chenna R, McGettigan PA, McWilliam H, Valentin F, Wallace IM, Wilm A, Lopez R, Thompson JD, Gibson TJ, Higgins DG. Clustal W and Clustal X version 2.0. Bioinformatics. 2007 Nov 1;23(21):2947-8. doi: 10.1093/bioinformatics/btm404. Epub 2007 Sep 10. PMID: 17846036.
